# Aging Affects Subcortical Pitch Information Encoding Differently in Humans With Different Language Backgrounds

**DOI:** 10.3389/fnagi.2022.816100

**Published:** 2022-04-13

**Authors:** Dongxin Liu, Jiong Hu, Songjian Wang, Xinxing Fu, Yuan Wang, Esther Pugh, Jennifer Henderson Sabes, Shuo Wang

**Affiliations:** ^1^Key Laboratory of Otolaryngology Head and Neck Surgery, Beijing Institute of Otolaryngology, Otolaryngology—Head and Neck Surgery, Ministry of Education, Beijing Tongren Hospital, Capital Medical University, Beijing, China; ^2^Department of Audiology, University of the Pacific, San Francisco, CA, United States; ^3^Department of Otolaryngology, Keck School of Medicine of USC, Los Angeles, CA, United States

**Keywords:** frequency following response (FFR), aging, language background, pitch coding, brainstem, pitch strength, pitch correlation

## Abstract

Aging and language background have been shown to affect pitch information encoding at the subcortical level. To study the individual and compounded effects on subcortical pitch information encoding, Frequency Following Responses were recorded from subjects across various ages and language backgrounds. Differences were found in pitch information encoding strength and accuracy among the groups, indicating that language experience and aging affect accuracy and magnitude of pitch information encoding ability at the subcortical level. Moreover, stronger effects of aging were seen in the magnitude of phase-locking in the native language speaker groups, while language background appears to have more impact on the accuracy of pitch tracking in older adult groups.

## Introduction

Speech is a stream of acoustic elements and the ability to decode these elements in a meaningful way is a complicated task involving complex neural processing (Chandrasekaran and Kraus, 2010). Relevant acoustic information must be encoded by temporal and spectral cues in subcortical structures and the signal sent to the auditory cortex (Eggermont, 2001; Hickok and Poeppel, 2007).

Pitch information encoding is part of the fundamental neural activities that occur at the subcortical level. Pitch-relevant neural activity in the auditory subcortical is not static, nor is it simply dedicated to faithfully reflecting the physical properties of the stimulus (Hall, 1979). Auditory nerve fibers demonstrate periodicities and inter-spike intervals that allow for the pitch information of complex speech to be encoded at the auditory subcortical level. These period-related cues relate to the fundamental frequency (F0) of the Frequency Following Response (FFR) (Miller and Sachs, 1984; Palmer et al., 1986). For example, in tonal language speakers, F0 features have been shown to provide dominant cues for high speech intelligibility of lexical tones, and a better understanding of subcortical and early cortical stages of perception (Krishnan et al., 2012). Scalp recorded FFR is thought to be generated from the inferior colliculus in animal models (Smith et al., 1975; Davis and Britt, 1984) and neurological data in humans (Bidelman, 2015), while the magnetoencephalography studies demonstrate that the auditory cortex also contributes (Coffey et al., 2016; Bidelman, 2018). Although many generators mentioned above may contribute to the origin of FFR, the nature of the auditory system makes it unlikely that the low-pass filtered phase-locked activity reflected in the FFR is of cortical origin (Akhoun et al., 2008). Therefore, FFR can be used as a window into the early stages of subcortical pitch processing, as well as an objective auditory electrophysiological assessment tool that has been increasingly used to assess synchronized neural activity and pitch information encoding (Russo et al., 2004; Chandrasekaran and Kraus, 2010). FFR has been viewed to be better at preserving spectrotemporal information related to complex sounds such as pitch in speech samples (Krishnan et al., 2004, 2005; Krishnan and Plack, 2011), as it faithfully follows the temporal and spectral characteristics of the stimulus. The encoding of pitch information preserved in the FFR is strongly correlated with perceptual measures (Krishnan et al., 2010a; Bidelman and Krishnan, 2011), suggesting that acoustic features related to pitch perception are well represented at the level of the brainstem. Many factors, such as language background, age, and music training, have been shown to impact various aspects of FFR (Russo et al., 2004; Clinard et al., 2010).

Hearing loss ranks third among chronic diseases in the elderly population (aged 65 and above) (Yueh et al., 2003). Three main factors are considered to contribute to age-related decline in speech perception, including peripheral hearing loss, central auditory processing deficits, and decreased cognitive function (Anderson and Karawani, 2020). However, it is difficult to completely separate contributions of peripheral hearing loss and aging factors as almost all older individuals have decreased hearing in high-frequency regions above 8,000 Hz (Davis et al., 1992; Matthews et al., 1997). In addition to peripheral hearing loss, aging may also reduce synchronization to sustained stimulus components and may degrade the processing of duration components of stimuli (Anderson and Karawani, 2020). Age-related decline has been found in temporal processing measured by gap detection test in humans (Schneider et al., 1994), and is known to affect auditory functions such as speech perception in noise and the FFR. Deficits in FFR in older adults appear to be related to speech perception deficits. Older adults, even with clinically normal hearing sensitivity, have auditory perceptual deficits compared to their younger counterparts (Fitzgibbons and Gordon-Salant, 1995; Pichora-Fuller et al., 1995; Gordon-Salant, 2006; Clinard et al., 2010; Wang et al., 2016). The physiological mechanism of auditory aging may be explained by decline in temporal processing (Clinard et al., 2010) including: neural inhibition (Caspary et al., 2005), neural firing, synchrony affected by temporal jitter (Pichora-Fuller and Schneider, 1992; Frisina and Frisina, 1997; Pichora-Fuller et al., 2007), prolonged neural recovery time (Walton et al., 1998), and decreased numbers of neurons in the auditory nuclei (Frisina and Walton, 2006). In FFR studies, it has been shown that older adults have reduced amplitudes and increased latency of waves in their FFRs (Wang et al., 2004; Anderson et al., 2012), suggesting lower phase locking ability and temporal processing capacity. It was suggested that age-related reduction in γ-aminobutyric acid (GABA) and glycine, which play an important role in neural processing of frequency modulations (Covey and Casseday, 1999), may interfere with older individual’s ability to phase lock to rapidly changing formants in the consonant transition (Anderson et al., 2012).

Experiences, such as language experience and musical training, may also shape the way the nervous system responds to sensory input and the subcortical pitch information encoding ability. The brain undergoes widespread neural specialization relating to language and cognitive processing (Costa and Sebastián-Gallés, 2014; Burgaleta et al., 2016). At the subcortical level, pitch processing of lexical tones can be shaped by language experience in both childhood and adulthood (Li et al., 2001; Wang et al., 2004; Liu et al., 2020). Previous studies between Mandarin speakers of tonal and non-tonal languages (English) have shown that pitch encoding at the subcortical level can be enhanced by experience in lexical tones, irrespective of speech or non-speech stimulus (Krishnan et al., 2005, 2009; Swaminathan et al., 2008). It was suggested that the reorganization of the brainstem pitch encoding mechanisms were shaped by the corticofugal system in the early stage of language development (Keuroghlian and Knudsen, 2007; Kral and Eggermont, 2007). It has also been shown that native speakers of non-tonal languages who learned Mandarin Chinese in their adulthood demonstrated weaker FFRs than native speakers of Mandarin Chinese, but enhanced subcortical neural pitch information encoding capacity compared to those that did not speak Mandarin Chinese at all (Liu et al., 2020). This young adult cohort was well past the “critical period” of language acquisition and exhibited greater pitch information encoding.

Lexical tones are continuous and curvilinear whereas in music, pitch unfolds in a discrete and stair-stepped manner (Dowling, 1978; Bidelman et al., 2011b). Though linguistic pitch patterns differ substantially from those used in music, long-term music training can enhance subcortical encoding of linguistic pitch patterns. FFR strength in response to band-pass-filtered harmonic complexes have shown to be enhanced by F0 discrimination training (Carcagno and Plack, 2011). It was shown that musicians and tonal language speakers have enhanced brainstem FFRs elicited by musical or linguistic pitch patterns (Bidelman et al., 2011a,b). Musicians have been shown to have better subcortical encoding of pitch in noise compared to non-musicians (Parbery-Clark et al., 2009; Bidelman and Krishnan, 2010; Chandrasekaran and Kraus, 2010), as musicianship may be able to modulate speech representations at multiple tiers of the auditory pathway, while strengthening the correspondence of processing between the subcortical and cortical areas (Bidelman, 2015). The neural mechanism governing experience-dependent plasticity is likely mediated by a coordinated interaction between the ascending and descending neural pathways (Chandrasekaran and Kraus, 2010). Animal studies (Suga et al., 2003) show that signal representation in subcortical structures may be modulated by the efferent corticofugal system and the enhanced subcortical activity in humans may also be mediated by the corticofugal system (Krishnan et al., 2005; Wong et al., 2007; Song et al., 2008).

These studies and others (Gao and Suga, 2000; Feng-Ming, 2017) have demonstrated how factors such as aging and language background influences one’s pitch information encoding as measured by FFR. However, none have shown the combined effect of the following factors: do individuals with tonal language background have less degradation in their subcortical pitch information encoding as they age? The current study is aimed at expanding on our previous work on the speech-evoked FFRs in individuals of different language experience and different ages, and to examine how different aspects of pitch information coding, such as strength of coding and the accuracy of coding, differ with different ages as well as language experiences. We hypothesize that younger tonal language speakers exhibit more robust and accurate subcortical encoding of lexical tones than older tonal language speakers, as well as non-tonal language speakers regardless of their ages. We also hypothesize that older tonal language speakers encode pitch information more accurately than their non-tonal language speaker counterparts.

## Materials and Methods

### Participants

Fifteen Chinese young (CHY) subjects (mean age ± SD = 24.1 ± 3.3 years; seven males and eight females), eleven Chinese older (CHO) subjects (mean age ± SD = 62.8 ± 3.0 years; four males and seven females), sixteen English young (ENY) subjects (mean age ± SD = 23.4 ± 3.1 years; 2 males and 14 females) and thirteen English older (ENO) subjects (mean age ± SD = 65 ± 3.2 years; 7 males and 6 females) were included in this study. CHY and CHO subjects were native speakers of Mandarin Chinese who were recruited at the Beijing Tongren Hospital in China. ENY and ENO subjects were native speakers of English recruited at the University of the Pacific in San Francisco, in the United States. All participants reported no neurological or otological symptoms or illness. They all had clinically normal hearing thresholds, defined as <25 dB HL at octave frequencies from 250 to 4,000 Hz. All participants presented normal immittance test results: Type A tympanograms and present ipsilateral and contralateral acoustic reflexes. To ensure normal peripheral hearing and intact integrity along the auditory pathway, click-evoked Auditory Brainstem Response (ABR) latencies were measured at 80 dB SPL, with a 100 μs click stimulus at a rate of 21.1 Hz. Wave V latencies of click-evoked ABRs were used as a benchmark for what would be considered clinically normal electrophysiological responses (no delay in wave V latencies). ABRs also serve as a quality control method for FFR recordings; a typical practice in previous works by present author(s) and others in the field (Wang et al., 2016; Liu et al., 2020). Wave Vs of ABR for all subjects were identified by experienced audiologists. Latencies of wave V from all subjects were measured at less than 6.5 ms, which is comparable to those reported in previous works (Anderson et al., 2012), suggesting clinically normal electrophysiological responses from all subjects. As discussed above, music training has been shown to impact FFR results. To avoid this, a modified version of the Munich Music (MUMU) Questionnaire (Frederigue-Lopes et al., 2015), which included four questions was used. As musical training has been shown to impact FFR responses from tonal language speakers and non-tonal language speakers (Bidelman et al., 2011a,b; Maggu et al., 2018), participants who answered “Yes” to prior musical training or vocal training were excluded to eliminate potential influence from long-term music training. All participants were provided written consent for this study, which was approved by the Institutional Review Board at the Beijing Institute of Otolaryngology, Beijing Tongren Hospital and University of the Pacific.

### Stimulus Parameters and Recording Procedure

Identical equipment, stimuli, and experiment protocols were used in the two collaboration sites to ensure data obtained the two sites were comparable. Detailed protocols and related quality control procedures can be found in previous collaborative work (Liu et al., 2020). Briefly, a monosyllabic Chinese word/yi/with high-falling tone, marked as/yi4/, was used as stimulus in this study. The voice sample was recorded from a male native speaker of Mandarin Chinese with a sampling rate of 40 kHz. Duration of the stimulus was set at 250 ms with 5 ms rise/fall time. The voice sample had a fundamental frequency (F0) trajectory of/yi4/, changed from 180 Hz to 130 Hz, with steady-state vowel formant frequencies at F1 = 400 Hz, F2 = 2,100 Hz, F3 = 3,000 Hz and F4 = 3,500 Hz. The stimulus was presented monaurally at 70 dB SPL at a repetition rate of 3.2/s.

Participants were asked to rest in a supine position with eyes closed. Gold-plated recording electrodes placed at the high forehead, low forehead and right mastoid acted as non-inverting recording, ground and inverting recording electrodes respectively. All impedances were kept ≤ 3 kΩ. FFR were recorded from each subject through an electromagnetically shielded insert earphone (ER-3A). Electroencephalogram (EEG) was collected using a single-channel recording with SmartEP system by Intelligent Hearing Systems (Miami, FL, United States). The sampling interval was fixed at 75 μs (recording sampling rate of 13,333 Hz). Bandpass filter was set between 100 Hz and 3,000 Hz. Recording sweeps containing any electrical activity exceeding 25 μV were rejected automatically and 2,000 artifact-free responses were collected. An additional click-evoked ABR was performed at the beginning and end of each recording session to ensure that the subjects had no adaptive responses.

### Response Evaluation and Data Analysis

To evaluate FFR data, the experiment was conducted in a passive listening paradigm. EEGs obtained from participants in all four groups were analyzed with customized Matlab scripts (Mathworks, Natick, MA, United States). A periodicity detection short-term autocorrelation algorithm (Boersma, 1993), which performs a short-term autocorrelation analysis on several small segments taken from the FFR and stimulus, was used to extract F0 contours from the FFR waveforms obtained from each participant.

Two main indexes were utilized in this study to quantitatively evaluate FFR. One index, Pitch Correlation, calculates the correlation coefficient between the stimulus’ F0 and the F0 extracted from each response. It examines the extent to which the F0s of the stimulus and FFR response are correlated. If the two F0 signals are identical, the cross correlation coefficient would be 1. If the two F0s are not correlated at all, the cross correlation coefficient would be 0 (Skoe and Kraus, 2010). Pitch Correlation is a useful index in studying the overall faithfulness of the “following” component of the FFR, reflecting the encoding accuracy of pitch (Hornickel et al., 2009).

The other index, Pitch Strength, is used to examine the robustness of periodicity contained in each FFR waveform. Pitch Strength is defined as the difference between the maximum and minimum autocorrelation coefficients, normalized between 0 and 1, of each FFR waveform. FFR waveforms contain periodic neural responses from the auditory subcortex elicited by the pitch information in the speech stimulus. Thus, Pitch Strength can be viewed as a representation of pitch salience or robustness reflecting neural synchrony and neural phase-locking ability (Krishnan et al., 2004).

As both Pitch Strength and Pitch Correlation range between 0 and 1 and were not strictly Gaussian, they then underwent a rationalized arcsine transform (Studebaker, 1985). RAU linearizes the proportional data (between 0 and 1) and converts them to Rational Arcsine Units (RAU), which is more suitable for linear statistical tests like ANOVA or *t*-test.

A two-way ANOVA was performed to test whether aging (young and older groups) and language background (Mandarin Chinese and English) have an interactive impact on Pitch Strength and Pitch Correlation. *Post hoc* multiple comparison tests were performed where applicable to examine the difference between groups. Statistical level of significance was set at *p* < 0.05.

## Results

### Temporal Waveforms and Spectrograms of Frequency Following Response

**FIGURE 1:**
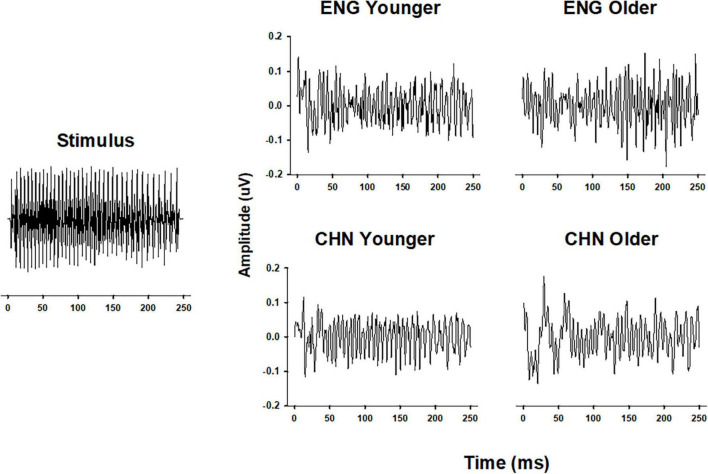
Comparison of FFR waveforms in different groups. Temporal waveforms of the original stimulus/yi4/(**left panel**) and of the grand-average FFR of all four groups (**right panel**). FFR waveforms were plotted as Amplitude (in μV) as a function of Time (in ms). ENY, English younger; ENO, English older; CHY, Chinese younger; CHO, Chinese older.

Grand-average temporal waveforms of FFRs for the four groups elicited by stimulus/yi4/are shown in Figure 1. Periodic components, which mimic those of the stimulus, can be observed in the temporal waveforms. Qualitatively, the group averaged FFRs from the CHY group had clearer and more stable periodicity, compared to those from the other groups.

**FIGURE 2:**
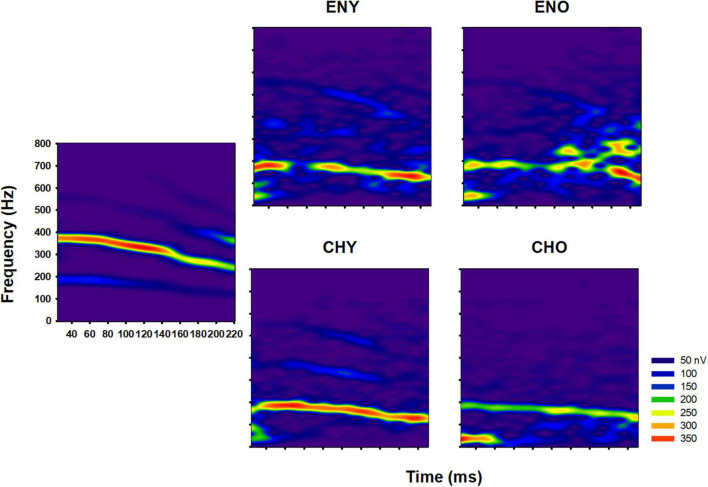
Comparison of FFR spectrograms in different groups. Spectrograms of the original stimulus/yi4/(**left panel**) and of the grand-average of FFR of all four groups (**right panel**). Spectrograms were plotted in Frequency (in Hz) as a function of Time (ms) and the colored heatmap represents spectral energy (nV).

Similarly, short-time spectrograms based on averaged FFR waveforms from the four groups are plotted in Figure 2. Colored heat scale in each spectrogram represents the level of spectral energy in the FFRs. Consistent with the temporal waveforms, qualitatively, the averaged F0 contours from CHY group had clear, continuous, and robust spectral energy in the stimulus’ F0 region, compared to those from the other groups.

### Statistical Analysis

**FIGURE 3:**
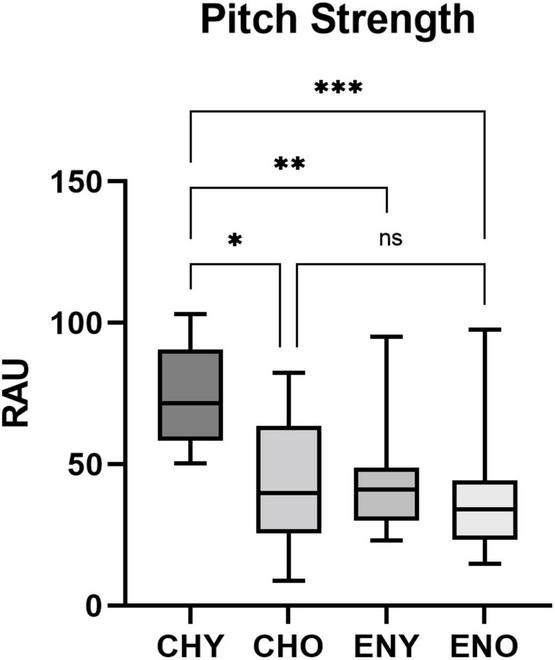
Pitch Strength of the four groups. Pitch Strength in RAU values obtained from all four groups, elicited by/yi4/. Boxes represent the interquartile ranges with whisker bars indicating the data ranges. Number of asterisks indicate levels of statistical significance between groups (*p* < 0.05, *p* < 0.01 and *p* < 0.001, respectively).

**FIGURE 4:**
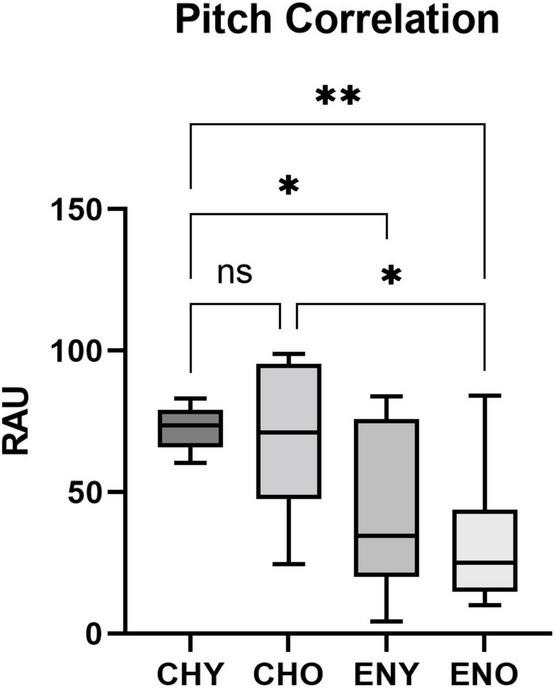
Pitch Correlation of the four groups. Pitch Correlation in RAU values obtained from all four groups, elicited by/yi4/. Boxes represent the interquartile range with whisker bars indicating the data ranges. Asterisks indicate statistical significance found in the factor of language experience (*p* < 0.0001, respectively).

Boxplots of the statistical data of Pitch Strength and Pitch Correlation were shown in Figures 3, 4.

### Two-Way ANOVA

For Pitch Strength homogeneity of variance across groups were verified by Levene Statics [*F*(3, 50) = 0.642, *p* = 0.592]. The interaction between age and language experiences revealed a significant effect on Pitch Strength in RAU [*F*(1,50) = 6.71, *p* = 0.026], with age [*F*(1,50) = 9.345, *p* = 0.004] and language experiences [*F*(1,50) = 11.77, *p* = 0.001], both having significant impact. *Post hoc* Tukey’s multiple comparison tests were carried out amongst all four groups (Table 1). Results revealed significant differences in Pitch Strength in RAU between the CHY vs, CHO, CHY vs. ENY, and CHY, and ENO groups.

**TABLE 1:** *Post hoc* group comparisons on pitch strength in RAU.

		CHY vs. CHO	CHY vs. ENY	CHY vs. ENO	CHO vs. ENY	CHO vs. ENO	ENY vs. ENO
Pitch strength (RAU)	Adjusted *P* value	0.0029*	0.0004**	0.0001***	0.9939	0.9463	0.8727

*Number of asterisks indicate levels of statistical significance between groups (p < 0.05, p < 0.01 and p < 0.001, respectively).*

For Pitch Correlation, heterogeneity of variance was observed [*F*(3, 50) = 7.955, *p* < 0.001]. It was evident that the CHY group, which had a highly “clustered” distribution of Pitch Correlation (Mean = 72.45, SD = 7.3), contributed to the heterogeneity of variance. When comparing variances of the CHO, ENY and ENO groups, homogeneity was confirmed [*F*(2, 36) = 0.438, *p* = 0.649]. Two-way ANOVA was still carried out. The interaction between age and language experience did not reveal a significant effect on Pitch Correlation in RAU [*F*(1,50) = 0.2126, *p* = 0.6468]. As for the two factors alone, age [*F*(1,50) = 1.137, *p* = 0.292] also did not have a significant effect, while language experience [*F*(1,50) = 26.47, *p* < 0.0001] had a significant impact on Pitch Correlation. *Post hoc* comparisons therefore were not carried out.

## Discussion

The present study compared Mandarin Chinese lexical syllable elicited FFRs obtained from native Chinese Mandarin speakers and native English speakers, both of various ages. Previously, studies have thoroughly documented the effects of aging on FFRs (Clinard et al., 2010; Anderson et al., 2012; Mamo et al., 2016; Presacco et al., 2019; Roque et al., 2019) and language background on FFRs (Krishnan et al., 2009, Krishnan et al., 2010a,^2012^; Bidelman et al., 2011a,b). Furthermore, these studies used either synthesized/da/or/a/to examine the effect of aging on non-tonal language speakers (Anderson et al., 2012; Presacco et al., 2019), or stimuli with various degrees of features from a tonal language as a speech token to examine the effect of language backgrounds on FFR. For example, Curvilinear Iterated Rippled Noises (IRN) in and out of Mandarin tonal space in Krishnan et al. (2009), and Music notes and time varying IRN were in Bidelman et al. (2011a). However, to our knowledge, the present study is the first with a relatively well controlled experiment protocol that enables the direct comparison of these two factors, using a linguistically and phonetically relevant natural speech token to elicit the FFR.

Our results showed that the CHY group had significantly better pitch information coding, both in magnitude and accuracy, compared to the other three groups. Considering previous studies on FFRs (Krishnan et al., 2005; Liu et al., 2020), this was not a surprising finding. More interestingly we demonstrated how different aspects of pitch information processing capacity, namely the magnitude and accuracy, may be influenced by the aging process and language experience in similar yet different ways.

### Language and Accuracy of Subcortical Pitch Information Coding

Pitch correlation, the index representing accuracy of pitch information coding, was found to be unaffected by the interaction between age and language background, but affected by language background alone. Pitch tracking accuracy in FFR influenced by language background is not a new revelation and is consistent with our previous report and other similar studies in the field (Krishnan et al., 2005; Liu et al., 2020). For example, a previous study (Krishnan et al., 2010c) found similar results suggesting that pitch features important to tone perception, are more resistant to degraded listening conditions among tonal language speakers than in non-tonal language speakers. Measurements of subcortical pitch encoding magnitude and accuracy were recorded and computed from young Chinese and young English participants. Iterated rippled noise (IRN) was used to mimic the degraded stimulus, preserving the perception of pitch but without the waveform periodicity or highly modulated stimulus envelopes. Similarly, in Bidelman et al. (2011a), when FFR was elicited by the IRN models based on Mandarin tone and major third music notes, the experience dependent plasticity (language and music training) was shown to enhance pitch tracking accuracy in groups with tonal language background as well as music training. These results suggested an adaptation of the experience-dependent brainstem mechanism in encoding and transmitting robust pitch relevant information. The differences we observed here between the two language groups in their pitch tracking accuracy was not surprising considering the life-long exposure of a tonal language in the CHY and CHO groups. In the CHO group, with the aging process, older Chinese speakers’ ability to accurately track pitch information did not appear to have deteriorated significantly, particularly when compared to the level of their English-speaking counterparts at the same age.

Subcortical pitch encoding can be considered a part of the temporal encoding strategy that is plastic and sensitive to language experience (Krishnan et al., 2005). This plasticity enhances neural activity of temporal intervals that carry acoustic and linguistic features of pitch information encoding. In their cross-language comparisons (Krishnan et al., 2010b), it was shown that non-tonal language speakers (English) had lower Pitch Strength than the tonal language speakers (Chinese and Thai) when using tonal stimulus (Mandarin and Thai tones) to record the FFR. Corticofugal influence is likely to explain this experience-dependent enhancement of the magnitude in pitch representation in tonal language speakers.

A possible explanation for the similarity and differences in accuracy of such plasticity is phase-locking ability potentially being enhanced by an excitatory and inhibitory neural interaction of pitch-relevant signal selections in the human auditory system (Ananthanarayan and Gerken, 1983). Different types of subcortical neurons subject to corticofugal egocentric selection may be sensitive to specific values of stimulus parameters (Krishnan et al., 2012). In our study, all Mandarin Chinese native speakers who have been exposed to the Mandarin Chinese language environment for decades, showed higher accuracy. It may be that the specific language environment requires more accurate pitch accuracy encoding ability in order to track lexical tones in their daily communications. This specific language experience and requirement may have, in some cases, introduced the corticofugal mechanism that may have resulted in enhanced neural sensitivity to pitch tracking.

Interestingly, in the comparison under our age × language framework, aging did not demonstrate significant influence on the accuracy of pitch encoding. Although the Mandarin Chinese speaking groups had more accurate pitch tracking neural responses than their English-speaking counterparts, age was not a factor for these groups (CHY vs. CHO). Accuracy of the CHO group’s subcortical pitch information coding did not appear to decline beyond that of their younger counterparts; this is in contrast to the significant differences in the magnitude of such ability between the two age groups, to be discussed later in this section.

It should be noted that the Pitch Correlation index obtained from the CHY group in this study revealed a highly “clustered” distribution of Pitch Correlation (Mean = 72.45, SD = 7.3). As a result, a heterogeneity of variance was observed during the two-way ANOVA analysis. Although the ANOVA analysis was still carried out, its results should be interpreted with caution.

### Aging and Magnitude of Subcortical Pitch Information Coding

Pitch strength, the index representing magnitude of pitch information coding, was found to be significantly affected by the interaction of both age and language backgrounds. Unsurprisingly, in the *post hoc* comparisons, the CHY groups’ pitch strength was significantly stronger than the CHO and ENO groups. These differences have been reported in multiple previous studies, as aging is associated with slower nerve conduction velocity (Peters, 2002) and decreased neural inhibition (Caspary et al., 2008), which results in age-related delays in neural transmission. FFR studies have typically found that older, non-tonal language speaking adults had weaker responses than younger adults. For example, Anderson et al. (2012) found that older English speakers had reduced phase-locking and response amplitudes for both transition and steady-state regions of the FFR when elicited by a 170 ms/da/. Similar results were observed in Presacco et al. (2019), where younger adults had stronger RMS in the steady-state region of their FFR than the older adults when the same/da/was used in conditions ranging from quiet down to 0 dB SNR.

The decline in the magnitude in encoding pitch information at the subcortical level may arise from age-related decreases in GABA inhibition, which has been seen in animal models (Caspary et al., 1995, 2005). Decline in inhibition function may result in degradation of subcortical temporal processing, contributing to age-related deficits in subcortical encoding of pitch and timing (Anderson et al., 2011).

Another theory that may explain why older adults have poorer speech perception is temporal jitter or neural noise in the auditory system (Pichora-Fuller et al., 2007; Wang et al., 2011). The loss of neural synchrony likely results in temporal jitter (Agmon, 2012; Luo et al., 2018), manifesting as the auditory system fails to generate synchronous firing to produce a precise representation of stimulus (Plack et al., 2014). In an experiment of mimicked neural jitter (Mamo et al., 2016), FFR was recorded in clean and jittered conditions to young (YNH) and older normal hearing listeners (ONH). The results showed that compared to the YNH listeners, the ONH listeners had significantly reduced magnitudes of F0 in clean condition, while in jittered condition, spectral magnitudes decreased only for the YNH listeners but not for the ONH listeners. These results can be attributed to the effects of aging on FFR, which are consistent with the results of the CHY and the CHO in our study.

Unlike the differences seen in tracking accuracy, in our study, CHO and ENO groups had significantly lower magnitudes in pitch information coding when compared to the CHY group, however, their magnitudes did not differ significantly from each other. While it appears the CHO group maintained a high accuracy in pitch tracking ability, the aging process may have contributed to a significant decline in magnitude of pitch tracking, consistent with previous reports, reducing their ability to levels comparable to their EHO peers, who likely had lower pitch tracking magnitudes to begin with, specifically as it pertains to the stimulus and set up used in this study.

### Language Experience and Aging May Affect the Accuracy and Magnitude of Subcortical Pitch Processing Capacity Differently

Not many FFR studies have looked at the combined effects of different factors such as language, aging, and music training on the magnitude and accuracy of subcortical pitch information encoding. One previous work (Maggu et al., 2018) attempted to examine the combined effect of language and music training on subcortical pitch encoding (Cantonese-speaking musicians vs. Cantonese-speaking non-musicians), where pitch encoding magnitude and accuracy were both measured when FFR was elicited by Cantonese tones and music notes. The authors found that while musical experience helps Cantonese-speaking musicians encode music notes with more precision, it does not further enhance their lexical tone encoding, neither in magnitude nor accuracy. It seems when two factors (in this case, language and music) are both known to enhance FFR, the combined effect is not an automatic further enhancement (Maggu et al., 2018) and is really an unknown territory and remains to be further explored.

In the present study, however, two factors are known to “counter” each other: tonal language background enhances the magnitude and accuracy of FFR, while aging weakens them. Our results showed that different age and language groups revealed unique patterns in the accuracy and magnitude of subcortical pitch information encoding when evaluated: with magnitude, the aging effects are seen only in the Chinese speaking groups, but not the English-speaking groups. However, a corresponding aging effect was not observed when accuracy was measured and compared. In contrast, with accuracy, it was clear that the effect of language backgrounds seems to separate groups by their language backgrounds and not their ages.

Traditionally, temporal processing, specifically phase-locking, has been considered to contribute primarily to the overall quality of the FFR, including the accuracy and the magnitude of the FFR (Krishnan et al., 2010a; Bidelman et al., 2011a; Skoe and Chandrasekaran, 2014). Our study suggests that although the aging process and language background both affect subcortical phase-locking capacity, they may do so differently. It may be that the decline in inhibitory function in the aging process may result in the degradation of subcortical temporal processing, contributing to the lack of robustness in phase-locking (Anderson and Karawani, 2020). On the other hand, specific features such as tones in Mandarin, with their functional relevance and importance to those native speakers, may have introduced the corticofugal mechanisms that results in enhanced neural sensitivity to pitch tracking (Krishnan et al., 2012). Such “feature-oriented” neural plasticity may be able to explain why the accuracy of pitch tracking remained higher in the CHO group in our study. Still, it is clear that under the age × language framework, how, and why, the magnitude and accuracy of subcortical pitch encoding are influenced by these factors require further study and exploration.

Lastly, it was observed that the ENY and ENO groups did not show any differences in accurately or robustly tracking pitch information at the subcortical level. This finding was somewhat different than those reported previously, where the older normal hearing English speakers showed reduced phase-locking than their younger counterparts (Anderson et al., 2012; Anderson and Karawani, 2020). One possible explanation could be that the stimulus used in the current study was a natural speech stimulus/yi4/, which is consistent with Krishnan’s study (Krishnan et al., 2010b). Unlike the widely used synthesized shorter/da/(Anderson et al., 2012),/yi4/possesses variations in pitch at the syllabic level, especially to the native speakers of Mandarin in this study. Language-dependent neuroplasticity occurs only when pitch in the auditory signal is part of the listener’s experience and relevant to speech perception, while a non-native pitch pattern fails to elicit a language-dependent effect (Krishnan et al., 2010b). It may be that corticofugal mechanisms induced by language experience plays a dominant role when a natural and native speech sample was used. Another small but notable factor could be that the stimulus used in the current study were presented at 70 dB SPL instead of 80 dB SPL as used in previous reports with the/da/stimulus. It may be that the lower intensity elicited a less robust and less accurate FFR in younger English speakers, while the older English speakers may not have had a robust or accurate FFR to begin with. In all, as suggested by Anderson and Karawani (2020), some previously observed trends in FFRs elicited by synthesized stimuli in aging populations may not hold when natural speech is used.

It should be noted that many aspects of FFR, including pitch encoding accuracy and magnitude, has been defined with slight variations in previous works on aging and/or language backgrounds. For example, FFR magnitudes were quantified by amplitude of FFT and SNR when tonal sweeps were used in young and older adults (Clinard and Cotter, 2015), and RMS amplitudes of various regions in the FFR waveforms when synthesized/da/was used (Anderson et al., 2012). Others have derived FFR magnitude from normalized autocorrelation functions when IRN (Krishnan et al., 2009; Bidelman et al., 2011b) or recorded tonal language samples (Wang et al., 2016; Liu et al., 2020) were used. Similarly, when discussing FFR accuracy, stimulus-to-response-correlation on the waveforms (Clinard and Cotter, 2015) or comparisons between stimulus f0 and response f0 have been used (Bidelman et al., 2011a; Xie et al., 2017; Liu et al., 2020). Differences in the definitions of these parameters call for further expansion of our current study to potentially include different stimuli or indexes used in previous studies. One of our future directions is to include tokens with fewer to no linguistic features to better examine the many layers of subcortical pitch information encoding in different populations. Regardless, further studies in this area are necessary.

## Conclusion

Numerous studies in related fields have examined how language backgrounds and the aging process may influence subcortical pitch information encoding. To our knowledge, however, the present study was the first in attempting to put these two factors in the same framework to examine the combined, and individual impact they have on FFR. It adds to the evidence that both language experience and aging are main factors, significantly affecting pitch information encoding ability at the subcortical level. We also demonstrate that accuracy and robustness may be different aspects of the FFR responses that can be influenced differently by aging and language experience. Some findings of this study are still relatively novel and somewhat different than previously reported. We plan to further expand our study to examine the underlying mechanisms of such differences and potentially shed light on the clinical utilization of these findings.

## Data Availability Statement

The raw data supporting the conclusions of this article will be made available by the authors, without undue reservation.

## Ethics Statement

The studies involving human participants were reviewed and approved by the Institutional Review Board at the Beijing Institute of Otolaryngology, Beijing Tongren Hospital and University of the Pacific. The patients/participants provided their written informed consent to participate in this study.

## Author Contributions

DL wrote the manuscript, performed statistical analysis, and collected data. JH collected data, introduced a new statistical method, edited, reviewed, and revised the manuscript. SW and XF collected the data. YW recruited the Chinese subjects and collected the data. EP edited, reviewed, and revised the manuscript. JH recruited the American subjects and collected the data. SW designed reviewed the manuscript and designed the research. All authors contributed to the article and approved the submitted version.

## Conflict of Interest

The authors declare that the research was conducted in the absence of any commercial or financial relationships that could be construed as a potential conflict of interest.

## Publisher’s Note

All claims expressed in this article are solely those of the authors and do not necessarily represent those of their affiliated organizations, or those of the publisher, the editors and the reviewers. Any product that may be evaluated in this article, or claim that may be made by its manufacturer, is not guaranteed or endorsed by the publisher.
